# Supplementary material for: Deep mutational scanning reveals the molecular determinants of RNA polymerase-mediated adaptation and tradeoffs
Source: Nat Commun. 2023 Oct 9;14:6319. doi: 10.1038/s41467-023-41882-7 (PMC10562459; doi:10.1038/s41467-023-41882-7)
Supplement: Supplementary file 1 — Supplementary Information [file 41467_2023_41882_MOESM1_ESM.pdf]

Supplementary Information

Title: Deep mutational scanning reveals the molecular determinants of RNA polymerase-mediated adaptation and tradeoffs

Supplementary Note 1: Validation of fitness and mutation diversity

We first validated the fitness scores using multiple methods. Each variants' fitness was estimated in biological replicate experiments. The fitness strongly correlated between biological replicate experiments (ρ = 0.87-0.99 across conditions and p-value < 10^{-16}) for all conditions and in all backgrounds.

Within each population, the same non-synonymous mutation occurred in combination with different synonymous mutations. For instance, the mutation F545L occurred alone and also as F545L + P560P, F545L + G585G, F545L + D549D etc. Since synonymous mutations usually do not alter protein function and fitness, such occurrences can serve as internal replicates for the non-synonymous mutations. In order to validate the fitness, we correlated the fitness between such internal replicates of the same mutation. Across conditions, the fitness of the internal replicates correlated strongly (ρ = 0.83-0.90 across conditions and p-value < 10^{-16}**).**

Additionally, mutations found in previous ALE experiments for improved growth in Glucose, Glycerol and NaCl had high fitness in our experiments (**Supplementary Table 1**). Mutations known to cause growth defects had a negative fitness in our analysis (**Supplementary Table 2**). Previously reported stringent mutations also had a high stringent enrichment score in our analysis (**Supplementary Table 3**). Finally, we also reconstructed and individually measured growth for multiple mutations. More information can be found subsequently in the text.

Mutations to residues with similar properties, such as leucine-isoleucine, may have fitness comparable to wild-type. Therefore, if certain positions had only such similar substitutions, their mean growth-associated fitness and mean stringent enrichment would be underestimated due to under-sampling. To verify the diversity of substitutions, we used the Grantham score, which is a measure of amino acid distance based on: composition, polarity, and molecular volume[1]. We mapped the residue-wise distribution of the Grantham score for all substitutions. We scored 5 +/-1 and 8 +/- 2 non-synonymous substitutions per position for growth and stringent enrichment respectively. We observed that substitutions at each position covered a broad distribution of Grantham scores at all positions. Additionally, the distribution of the Grantham score was comparable between stringent versus non-stringent and growth-promoting versus non-growth-promoting residues (**Supplementary Figure S1E and S1F)**.

Supplementary Note 2: Epistasis and means to determine interactions

Epistasis occurs when the actual fitness of a double mutant deviates from (is greater than or lower than) the sum of fitness of individual mutations. If we have mutations A, B and their combination AB, and fitness associated with each mutation is f_A, f_B and f_{AB} then epistasis is measured as:

$$\varepsilon_{AB} = f_{AB} - (f_A + f_B)$$

if $\varepsilon_{AB} > 0$, then the epistasis is positive and when $\varepsilon_{AB} < 0$, then the epistasis is negative. Epistasis between two residues usually suggests interactions. However, in the case of negative epistasis, a negative impact of combining two mutations on the function could be also due altered protein stability because two mutations are more likely to affect protein stability as compared to one. However, Positive epistasis i.e., cases where the actual fitness in greater than the sum of fitness is a more conclusive result of functional interactions. In the figures, 4 and 5, we only demonstrate significant positive epistasis.

We determined the significance of the epistatic interaction by using the combination of synonymous mutations as a control. Usually, a combination of synonymous mutations should not have any functional hypothesis. As expected the distribution of ε_{AB} for combination of synonymous mutations centered around 0 (**Figure S4**). Therefore, a distribution of ε_{AB} for synonymous mutations gives us the range for a null hypothesis: an absence of epistasis. Only ε_{AB} values greater than mean (ε_{AB} for combination of synonymous mutations) + 1.96 * (standard deviation of ε_{AB} for combination of synonymous mutations) to obtain a $p < 0.05$, we considered as significant (**Figure S4** for the range).

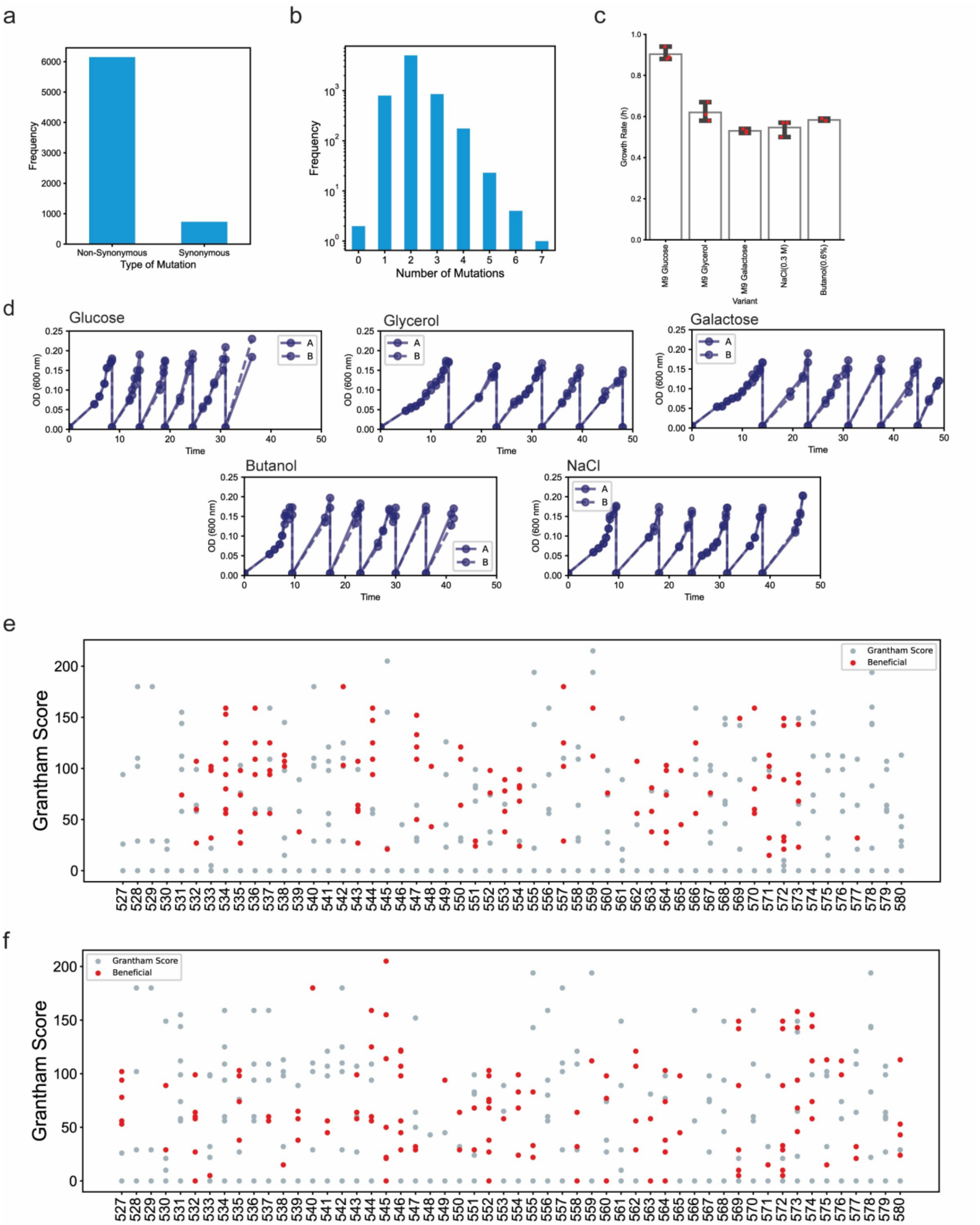

Supplementary Fig. 1: Mutation library statics and evolution experiment:

a. Frequency of non-synonymous and synonymous mutations within the library. A variant is considered non-synonymous if at least one of the mutations in the variant is non-synonymous. A variant is considered synonymous if all mutations are synonymous.
b. Histogram of number of mutations per variants.
c. Growth rate of *Escherichia coli* MG1655 strain in the multiple evolution environments from left to right: M9 minimal media with Glucose, M9 minimal media with Glycerol, M9 minimal media with Galactose, M9 minimal media with Glucose and 0.3M NaCl, M9 minimal media

with Glucose and 0.6% (v/v) butanol. Bars represent mean +/- SD associated with n = 3 replicates.

d. In each environment, the evolution was performed for 30 generations. Bottles with each media were inoculated to an initial OD of 0.005. When the optical density reached and OD (600 nm) of 0.18-0.2, the cells were inoculated in a new bottle with fresh media at a 1:32 dilution. In the figure, each blue line represents the tracking of the optical density in each cycle. The evolution was performed in two biological replicates A (solid line) and B (dashed line).

e. A position-wise distribution of Grantham score for all (gray) and beneficial mutations (red) for substitutions scored in the stringent selection. (The Grantham score measures the distance of a protein substitution based on chemical properties based on composition, polarity and molecular volume.

f. A position-wise distribution of Grantham score for all (gray) and beneficial mutations (red) for substitutions scored in the growth selection. (The Grantham score measures the distance of a protein substitution based on chemical properties based on composition, polarity and molecular volume.

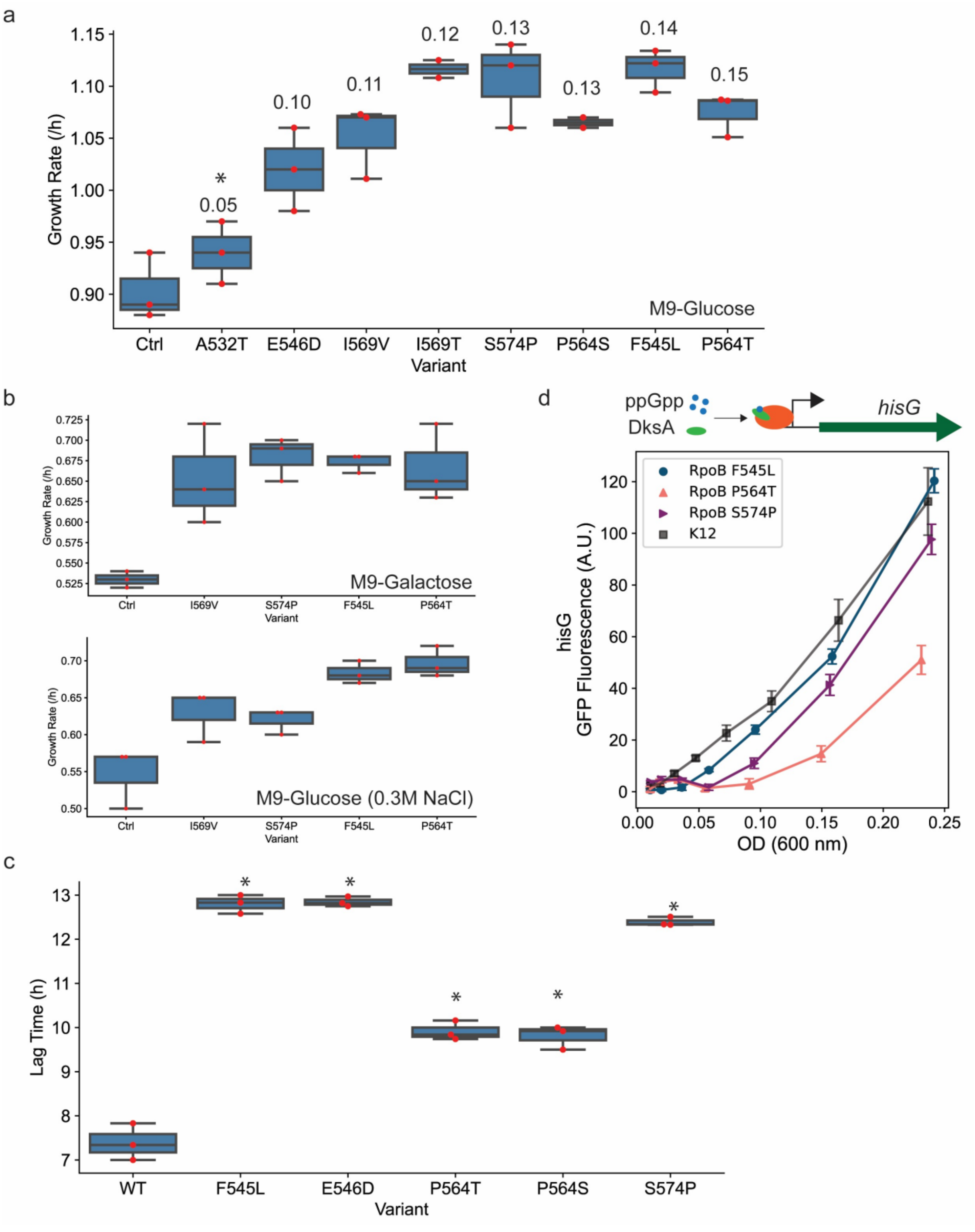

Supplementary Fig. 2: Target mutations alter global traits

Box plot comparing growth rate for eight different variants of RpoB (with high fitness across all 5 tested environments) and wild type cells, measured in

a. M9 minimal media with Glucose and

g. B) M9 minimal media with Galactose (top) and M9 Glucose with 0.3 M NaCl (Bottom). The value on top of each boxplot is the fitness measured in the CREPE evolution experiment. Each box plot represents a distribution of growth rates measured in n = 3 biological replicates. Boxes represent mean +/- SD associated with n = 3 replicates.

b. Box plot comparing lag for five growth-improving mutations for a switch between glucose and acetate to wild type cells. Each box plot represents lag measured in n = 3 biological replicates. * indicates significance with $p < 0.05$ using a Student's t-test.
c. Each curve represents the expression from *hisG* gene measured in fluorescence units using a fluorescent GFP reported under the *hisG* promoter at different optical densities of the culture. We measured the expression for the wild-type cells (black squares), the RpoB F545L mutant (Blue spheres), the RpoB P564T mutant (pink straight triangle), and the RpoB S574P mutant (purple inverted triangle). Each point represents the average fluorescence for three technical replicates with the error bard represents the standard deviation of the three replicates. It is important to note here that all fluorescence measurements were done in M9 minimal media and glucose supplemented with Cas amino acids. In this media, the growth rate for the mutants was comparable.

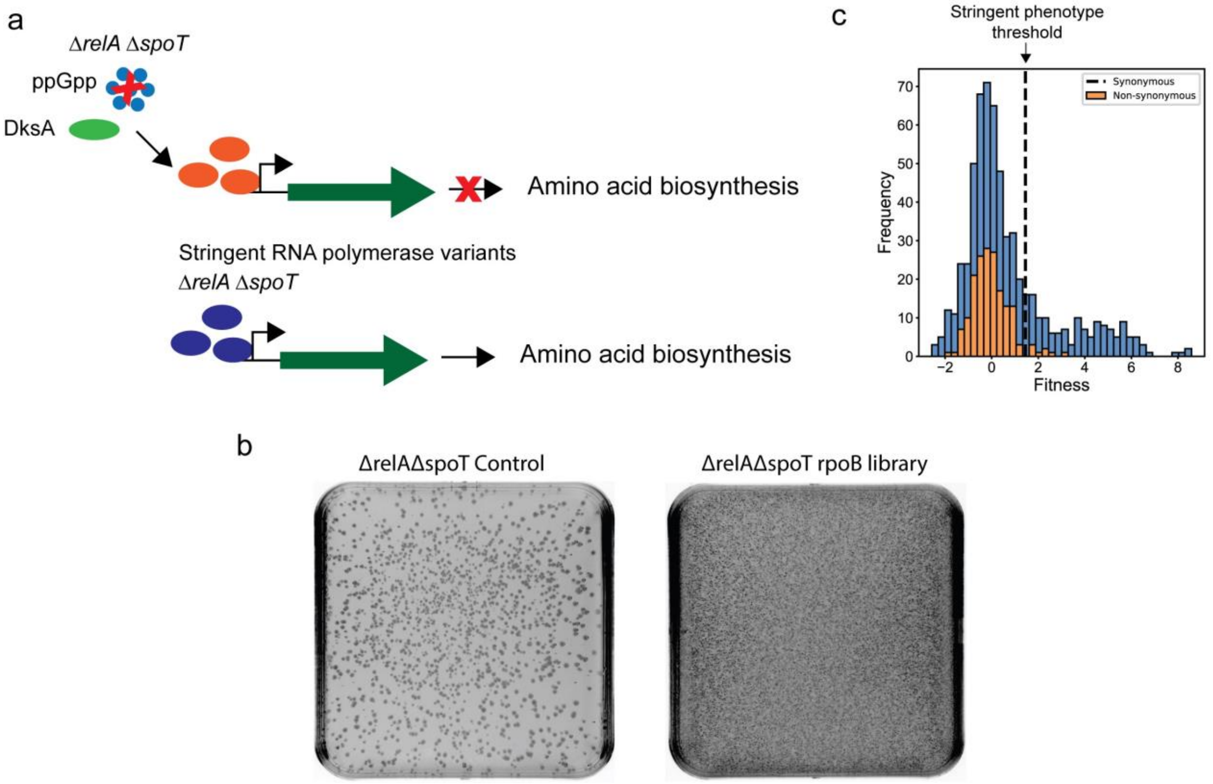

Supplementary Fig. 3: Selection for stringent mutations of the RNA polymerase

a. In the Δ*relA*Δ*spoT* strain of E coli, the wild type RNA polymerase cannot activate amino acid biosynthesis, while the stringent mutations of the RNA polymerase can.
b. A comparison of the colony forming units (CFUs) when 10^8 cells each for Δ*relA*Δ*spoT* strain of *E. coli* with wildtype RNAP (left) and Δ*relA*Δ*spoT* strain of *E. coli* with a variant library in the target region were plated on M9 minimal media without any amino acids.
c. Distribution of enrichment for different mutations estimated as: the log change in the frequency of the RpoB variant in the Δ*relA*Δ*spoT* background before and after plating on M9 minimal media. The Blue distribution represents all target variants and the orange histogram includes only synonymous variants of the RNA polymerase. The cut-off to determine if a variant is stringent is determined by the black line. The cut-off was set as an Enrichment score >= mean enrichment score for synonymous variants + 2.56 * standard deviation of the enrichment score for synonymous variants (measured using bootstrapping, Material and methods, and Supplementary code).

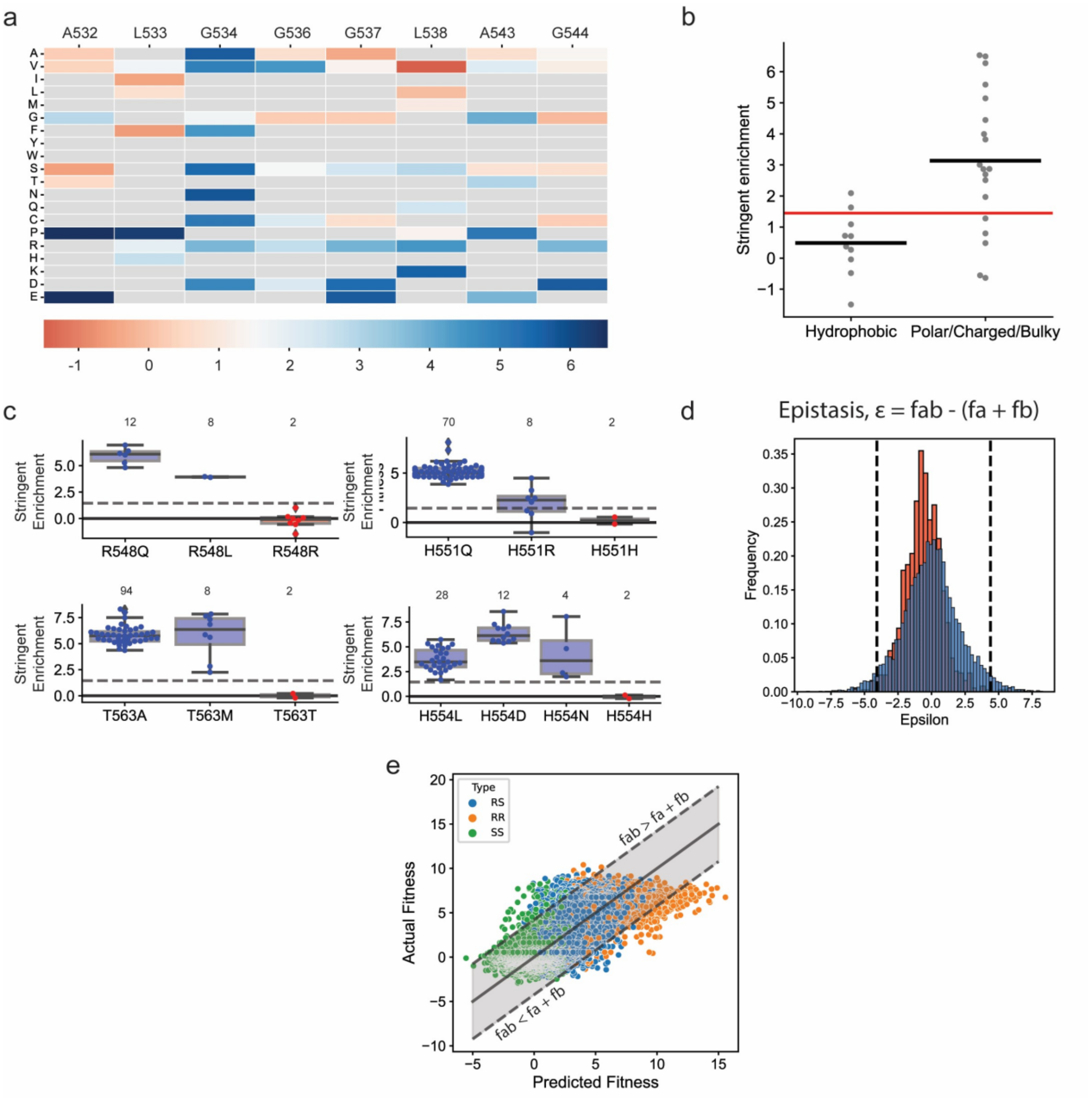

Supplementary Fig. 4: Characterizing stringent mutations of the target and epistasis associated with their combination:

a. bar plot (blue) represents stringent enrichment score of mutations in the polar residues that lead to loss of the internal interaction and the red bar represents synonymous mutation at the same position. The blue and red bars represent mean enrichment score of all internal replicate mutations from two biological replicate mutations.
b. A category plot comparing the stringent enrichment scores for mutations to hydrophobic residues to those of residues with polar, charged, and bulky residues. The red line represents the threshold for beneficial mutations. The data compares means for n = 10, and 19 measurements for Hydrophobic and Polar/Charged/Bulky changes respectively.
c. A bar plot (blue) of stringent enrichment score of non-synonymous (blue) and synonymous (red) mutations for some highlighted residues. Within each box, the horizontal black lines represent median values, lower and upper bounds correspond to the 25th and 75th percentile, and the whiskers extend to the extreme values within the 1.5 x interquartile range. They were n independent observations indicated for each variant in the plot. An observation represents an independent fitness measurement of a synonymous variant of the focal mutation in two biological replicates

d. Epistasis (ε) is measured as the difference between the fitness of a double mutant (fab) and the sum of fitness of individual mutants (fa + fb) (top). The histogram represents a distribution of ε for the combination of two non-synonymous mutations (blue) and the combination of two synonymous mutations (red, as a control). The black lines represent the cutoff for significant epistasis, determined as ε value greater than mean ε (synonymous) + 1.96*standard deviation ε (synonymous) (measured using bootstrapping, Material and methods, and Supplementary code).
e. A comparison of actual fitness (fab) and predicted fitness (fa+fb) for double mutants of the RNA polymerase. RR, RS, and SS represent combination of both stringent, one stringent and one non-stringent and both non-stringent individual mutations respectively. The gray zone represents the region of no epistasis.

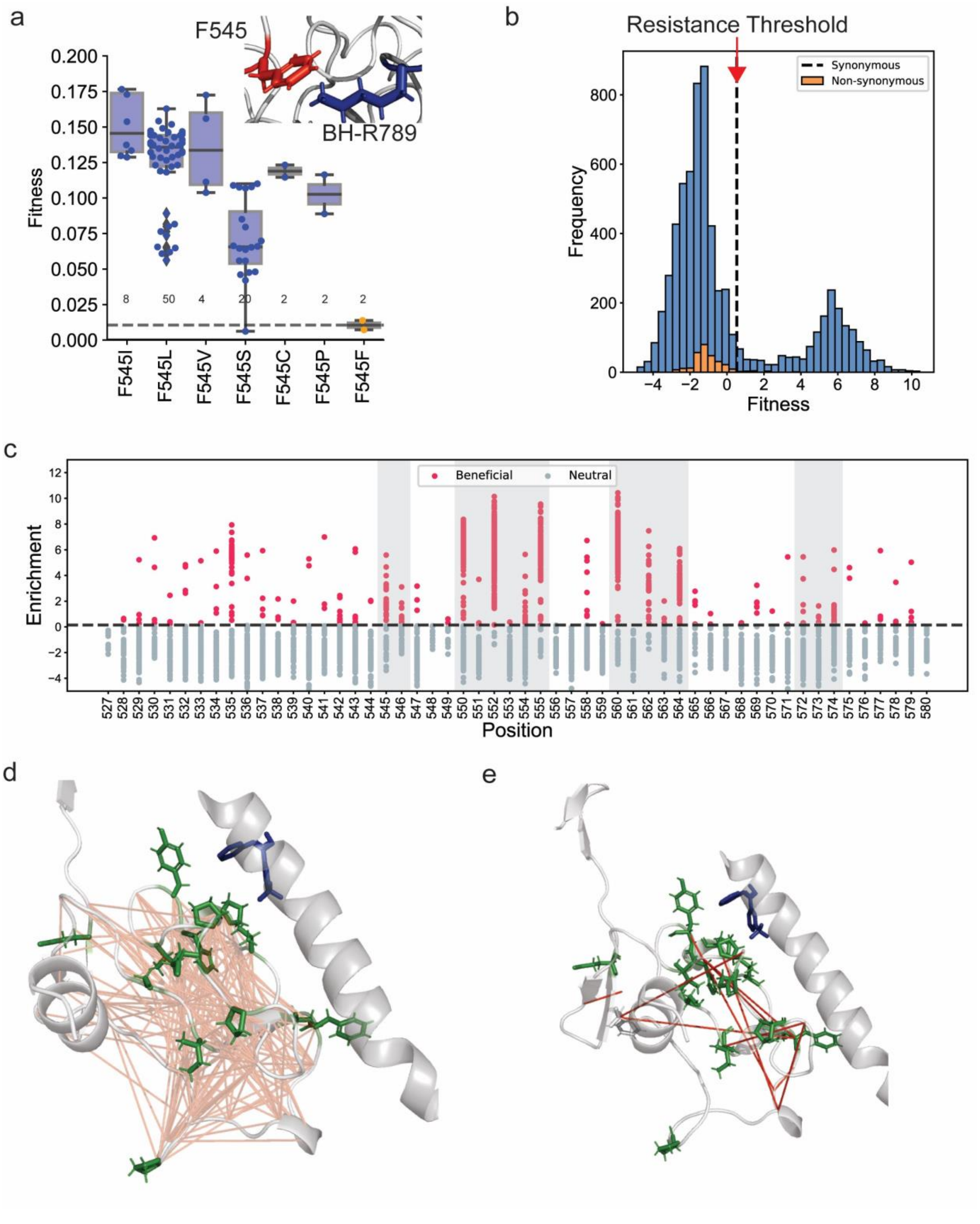

Supplementary Fig. 5: Characterizing growth-improving mutations of the target and epistasis associated with their combination:

a. (Inset) The residue F545 occurred in close proximity to a lysine, K789, residue. Aromatic rings in close proximity to positively charged residues can form cation-pi interactions. A bar plot (blue) of mean growth-associated fitness for substitutions to amino acids with positively charges (blue) and negatively charged (red) sidechains. Within each box, the horizontal black lines represent median values, lower and upper bounds correspond to the 25th and 75th percentile, and the whiskers extend to the extreme values within the 1.5 x interquartile range. The top and bottom dashed lines represent the cutoff for beneficial and deleterious fitness scores

respectively. They were n independent observations indicated for each variant in the plot. An observation represents an independent fitness measurement of a synonymous variant of the focal mutation in two biological replicates.

b. Distribution of enrichment for different mutations estimated as: the log change in the frequency of the RpoB variant in the Δ*tolC* background before and after plating on LB agar with 8 µg/mL of CBR703. The blue distribution represents all target variants and the orange histogram includes only synonymous variants of the RNA polymerase. The cut-off to determine if a variant is resistant to CBR703 is determined by the black line. The cut-off was set as an enrichment score >= mean enrichment score for synonymous variants + 2.56 * standard deviation of the enrichment score for synonymous variants (measured using bootstrapping, Material and methods, and Supplementary code).

c. Residue-wise distribution of enrichment values for the selection for growth in the *ΔtolC* background. The line represents the cut-off for the CBR703 resistant phenotype. Each dot represents an individual variant by position. Variants (red) above the cut-off are stringent mutations of the RNA polymerase.

d. Residues with high growth-associated fitness on the structure of the target region (green sticks). The red lines connect two residues with significant epistatic interaction for CBR703 selection **B)** for growth-associated fitness.

e. Residues with high growth-associated fitness on the structure of the target region (green sticks). The red lines connect two residues with significant epistatic interaction for growth-associated fitness.

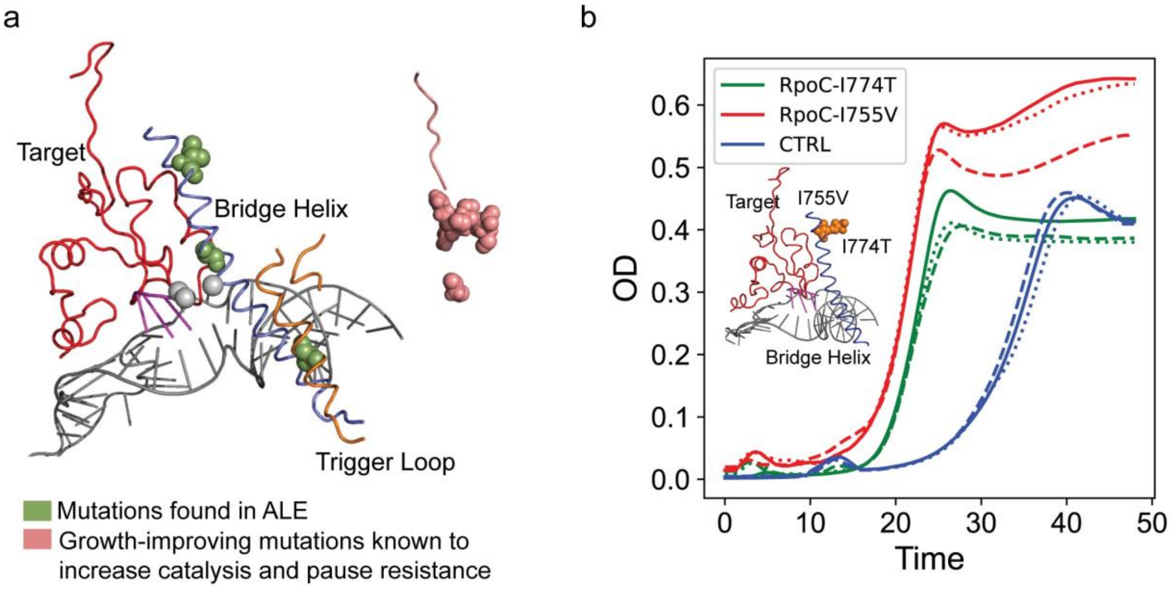

Supplementary Fig. 6: Growth may be determined by catalysis rate and pause resistance

a. Mutations found in ALE for improved growth (grey and green spheres) cluster around the BH and Trigger loop regions of the RNA polymerase known to control catalysis. Mutations known to impacr catalysis rate and pause resistance (substitutions in pink spheres and deletion of the pink cartoon loop region) also improve growth.
b. (Inset) Mutations RpoC subunit of the RNAP I755V and I774T in and close to the BH respectively are known to increase catalysis and pause resistance. (Graph) Comparison of growth of RpoC mutant I755V (red), RpoC mutant I774T (green), and wild type control (blue) in biological triplicates (represented with straight, dashes and dotted lines respectively).

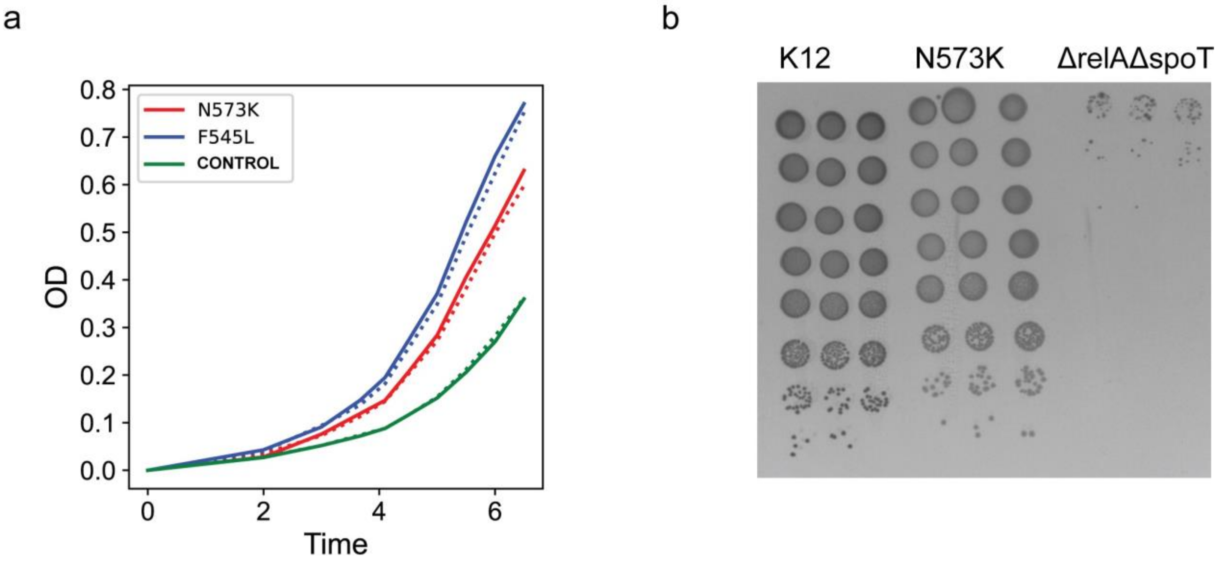

Supplementary Fig. 7: Mutation N573K leads to loss of both interactions:

a. Comparison of growth of RpoB growth-improving mutant F545L (blue), N573K (red), and wild type control (green) in two biological replicates (represented with straight and dotted lines respectively).
b. Dilution plating of on Wild type K12, RpoB N573K, and Δ*relA*Δ*spoT* strains on M9 minimal media with Glucose without supplementing amino acids. The dilution increased from 10^0 to 10^{-7} from top to bottom.

Supplementary Table 1: Mutations found in previous ALE experiments with the corresponding fitness score measured using CREPE in M9 minimal media + Glucose

S No	Mutation	Stress	Growth Media	Fitness Glucose	Reference
1	I569L	Osmolarity	M9 Minimal	0.12	[2]
2	E546V	Glucose	M9 Minimal	0.14	[3]
3	E562V	Glycerol	M9 Minimal	0.12	[3]
4	E562V	Glycerol	M9 Minimal	0.12	[3]
5	E546K	Glycerol	M9 Minimal	0.1	[3]
6	E546A	Glycerol	M9 Minimal	0.09	[3]
7	H526Y	Silver Nanoparticles	M9 Minimal	0.072	[4]
8	H526Y	Antibiotics	M9 Minimal	0.072	[5]
9	T539P	Temperature	M9 Minimal	0.047	[6]
10	G544S	Antibiotics	M9 Minimal	0	[7]
11	T553I	Temperature	Davis Media	-0.04	[6]
12	G556S	Temperature	Davis Media	-0.024	[6]
13	I572F	Temperature	Davis Media	-0.05	[6]
14	I572L	Temperature	Davis Media	0.01	[6]

Supplementary Table 2: Previously reported stringent mutations of the RNA polymerase and their corresponding Enrichment scores calculated using CREPE. The threshold for the stringent mutations is a enrichment score of 1.45.

**S. No.**	**Mutation**	**Study**	**Enrichment Score A**	**Enrichment Score B**
1	A532E	Modulation of DNA repair by mutations flanking the DNA channel through RNA polymerase	6.5	6.7
2	L533P		6.3	6.2
3	G534C		5.1	5
4	G536V		4.5	4.5
5	G537D		5.3	5.2
6	V550E		3.8	3.8
7	H551P		1.8	2
8	T563P		2.5	2.7
9	L571Q		5.6	5.6
10	I572S		5.2	5.2

Supplementary Table 3: Previously reported growth defects for different stringent mutations of the RNA polymerase

**S. No.**	**Growth Media**	**Mutation**	**Growth Rate/ Doubling Time**	**Glucose Fitness Replicate A**	**Glucose Fitness Replicate B**	**Reference**
1	M9 Minimal + Glucose	WT	40 min			[8]
2		S522F	48 min	-0.05961	-0.07499	[8]
3		I572F	48 min	-0.249201	-0.253652	[8]
4	DM Minimal + Glucose	WT	1.288 (/h)			[9]
5		I572F	0.898 (/h)	-0.249201	-0.253652	[9]

Supplementary Table 4: Single and multiple regression analysis of fitness with respect to DNA distance and RpoC distance. Statistics for single regression analysis were performed using Pearson Correlation estimates. Statistics for multiple regression analysis were performed using Multiple linear regression with p-value indicating the probability that an uncorrelated system produces the same Pearson correlation at least as extreme as the one computed from our datasets.

	**Pearson Correlation**		**Multiple Regression Analysis**	
	**RpoC-Distance**	**DNA-Distance**	**RpoC-Distance**	**DNA-Distance**
Growth	*Coefficient: -0.3*<br>*p-value* $< 10^{-7}$	*Coefficient: -0.04*<br>*p-value = 0.4*	*p-value < 0.05*	*p-value = 0.31*
Stringent	*Coefficient: -0.04*<br>*p-value = 0.24*	*Coefficient: -0.35*<br>*p-value* $< 10^{-7}$	*p-value = 0.34*	*p-value < 0.05*

Supplementary Table 5: List of primers and DNA sequences:

**Name**	**Sequence**	**Notes**
**F_rpoB_W1**	gtatcctgagcaaagacgacatcattg	Genome ampilifciation of rpoB window
**R_rpoB_W1**	caacagcacgttccataccagtac	Genome ampilifciation of rpoB window
**F_pSAH_rpoB_W1**	gataagccgctggttggtactggtatggaacgtgctgttgAGCGAGAGACGGACACGAAC	Backbone ampilifciation to clone rpoB window
**R_pSAH_rpoB_W1**	tttttcataacatcaatgatgtcgtctttgctcaggatacGGACAGAGACGGCTGAGGTG	Backbone ampilifciation to clone rpoB window
**rpoB1_mut_f**	caacccgctgtctgagatta	Error-prone PCR of rpoB window
**rpoB1_mut_r**	gcagacaggtagtgaatttc	Error-prone PCR of rpoB window
**rpoB_nextgenseq_for**	TCGTCGGCAGCGTCAGATGTGTATAAGAGACAGNNNNNNcaacccgctgtctgagatta	Next-gen sequencing of *rpoB* library
**rpoB_nextgenseq_rev**	GTCTCGTGGGCTCGGAGATGTGTATAAGAGACAGNNNNNNgcagacaggtagtgaatttc	Next-gen sequencing of *rpoB* library
**galK gRNA**	atgataaagctgctgcaata	gRNA spacer to manipulate *galK*
**rpoB gRNA**	accgatgttcggaccttcagg	gRNA spacer to manipulate *rpoB*

Supplementary References:

1. Grantham, R. Amino acid difference formula to help explain protein evolution. *Science* **185**, 862–864 (1974).
2. Dragosits, M., Mozhayskiy, V., Quinones-Soto, S., Park, J. & Tagkopoulos, I. Evolutionary potential, cross-stress behavior and the genetic basis of acquired stress resistance in Escherichia coli. *Mol. Syst. Biol.* **9**, 643 (2013).
3. Utrilla, J. *et al.* Global Rebalancing of Cellular Resources by Pleiotropic Point Mutations Illustrates a Multi-scale Mechanism of Adaptive Evolution. *Cell Syst* **2**, 260–271 (2016).
4. Graves, J. L., Jr *et al.* Rapid evolution of silver nanoparticle resistance in Escherichia coli. *Front. Genet.* **6**, 42 (2015).
5. Toprak, E. *et al.* Evolutionary paths to antibiotic resistance under dynamically sustained drug selection. *Nat. Genet.* **44**, 101–105 (2011).
6. Tenaillon, O. *et al.* The molecular diversity of adaptive convergence. *Science* **335**, 457–461 (2012).
7. Thulin, E., Sundqvist, M. & Andersson, D. I. Amdinocillin (Mecillinam) resistance mutations in clinical isolates and laboratory-selected mutants of Escherichia coli. *Antimicrob. Agents Chemother.* **59**, 1718–1727 (2015).
8. Jin, D. J. & Gross, C. A. Characterization of the pleiotropic phenotypes of rifampin-resistant rpoB mutants of Escherichia coli. *J. Bacteriol.* **171**, 5229–5231 (1989).
9. González-González, A., Hug, S. M., Rodríguez-Verdugo, A., Patel, J. S. & Gaut, B. S. Adaptive Mutations in RNA Polymerase and the Transcriptional Terminator Rho Have Similar Effects on Escherichia coli Gene Expression. *Mol. Biol. Evol.* **34**, 2839–2855 (2017).
