# Supplementary material for: Deep mutational scanning reveals the molecular determinants of RNA polymerase-mediated adaptation and tradeoffs
Source: Nat Commun. 2023 Oct 9;14:6319. doi: 10.1038/s41467-023-41882-7 (PMC10562459; doi:10.1038/s41467-023-41882-7)
Supplement: Supplementary file 3 — Reporting Summary [file 41467_2023_41882_MOESM3_ESM.pdf]

nature portfolio

Corresponding author(s): Olivier Tenaillon

Last updated by author(s): Sep 5, 2023

Reporting Summary

Nature Portfolio wishes to improve the reproducibility of the work that we publish. This form provides structure for consistency and transparency in reporting. For further information on Nature Portfolio policies, see our Editorial Policies and the Editorial Policy Checklist.

Statistics

For all statistical analyses, confirm that the following items are present in the figure legend, table legend, main text, or Methods section.

n/a	Confirmed	
☐	☒	The exact sample size (*n*) for each experimental group/condition, given as a discrete number and unit of measurement
☐	☒	A statement on whether measurements were taken from distinct samples or whether the same sample was measured repeatedly
☐	☒	The statistical test(s) used AND whether they are one- or two-sided<br>*Only common tests should be described solely by name; describe more complex techniques in the Methods section.*
☒	☐	A description of all covariates tested
☐	☒	A description of any assumptions or corrections, such as tests of normality and adjustment for multiple comparisons
☐	☒	A full description of the statistical parameters including central tendency (e.g. means) or other basic estimates (e.g. regression coefficient) AND variation (e.g. standard deviation) or associated estimates of uncertainty (e.g. confidence intervals)
☐	☒	For null hypothesis testing, the test statistic (e.g. *F*, *t*, *r*) with confidence intervals, effect sizes, degrees of freedom and *P* value noted<br>*Give P values as exact values whenever suitable.*
☒	☐	For Bayesian analysis, information on the choice of priors and Markov chain Monte Carlo settings
☒	☐	For hierarchical and complex designs, identification of the appropriate level for tests and full reporting of outcomes
☐	☒	Estimates of effect sizes (e.g. Cohen's *d*, Pearson's *r*), indicating how they were calculated

Our web collection on statistics for biologists contains articles on many of the points above.

Software and code

Policy information about availability of computer code

Data collection	The sequencing was performed using paired-end 2X150 np sequencing on the Illumina NextSeq platform. All sequencing data has also been submitted on ENA under the project accession number PRJEB59215.
Data analysis	We wrote custom code to analyze the data: https://datadryad.org/stash/share/ZfUDt7kbGXPGw4_tl_roBQ2okJde3oKFftqgAwYlF0o. Within the code we used custom packages: Python 3.7 and packages within Python SciPy, SciKitLearn, Numpy, and Pandas. For all structural analysis we used Pymol version 4.6 and Build 30.0.101.1338. All details are in the methods section as well. All code used to analyze the data can be accessed using the Zenodo link: https://zenodo.org/record/8034094

For manuscripts utilizing custom algorithms or software that are central to the research but not yet described in published literature, software must be made available to editors and reviewers. We strongly encourage code deposition in a community repository (e.g. GitHub). See the Nature Portfolio guidelines for submitting code & software for further information.

Data

Policy information about availability of data

All manuscripts must include a data availability statement. This statement should provide the following information, where applicable:

- Accession codes, unique identifiers, or web links for publicly available datasets
- A description of any restrictions on data availability
- For clinical datasets or third party data, please ensure that the statement adheres to our policy

All sequencing data has also been submitted on ENA under the project accession number PRJEB59215. The sequencing data can also be accessed at DRYAD database: https://datadryad.org/stash/share/pMgkxJauRphu1eU49XyxLOznG6zakpHaWBgLqzCcL-Y. The processed data can be accessed using the Dryad link: https://datadryad.org/stash/share/ZfUDt7kbGXPGw4_tl_roBQ2okJde3oKFftqgAwYlF0o. Source data has been provided with the paper.

Human research participants

Policy information about studies involving human research participants and Sex and Gender in Research.

Reporting on sex and gender	N/A
Population characteristics	N/A
Recruitment	N/A
Ethics oversight	N/A

Note that full information on the approval of the study protocol must also be provided in the manuscript.

Field-specific reporting

Please select the one below that is the best fit for your research. If you are not sure, read the appropriate sections before making your selection.

☒ Life sciences ☐ Behavioural & social sciences ☐ Ecological, evolutionary & environmental sciences

For a reference copy of the document with all sections, see nature.com/documents/nr-reporting-summary-flat.pdf

Life sciences study design

All studies must disclose on these points even when the disclosure is negative.

Sample size	Within each experiment, we characterized ~6000 mutations. The fitness for each variant in each condition was measured in biological replicate experiments. Within each population, the same non-synonymous mutation occurred in combination with different synonymous mutations. For instance, the mutation F545L occurred alone and also as F545L + P560P, F545L + G585G, F545L + D549D etc. Since synonymous mutations usually do not alter protein function and fitness, such occurrences can serve as internal replicates for the non-synonymous mutations. The fitness between such replicates was strongly correlated (ρ = 0.83-0.90 across conditions and p-value < 10-16). When making comparison using for a single variant the sample size was estimated as the number of times the fitness measured in each biological replicate experiment for all synonymous variants. When we considered populations with a particular behavior, the sample size was determined using a cut-off for the behavior. For instance, we use synonymous mutations as a control for wild-type behaviour. So, mutations with fitness more than 2.56*standard deviation more than the mean of synonymous mutations were conidered be beneficial. So, sample sizes were estimated as the variants meeting this criteria.
Data exclusions	No data were excluded from the analysis.
Replication	All experiments were done in two Biological replicates. The fitness scores was also validated using several internal biological replicates. Within each population, the same non-synonymous mutation occurred in combination with different synonymous mutations. For instance, the mutation F545L occurred alone and also as F545L + P560P, F545L + G585G, F545L + D549D etc. Since synonymous mutations usually do not alter protein function and fitness, such occurrences can serve as internal replicates for the non-synonymous mutations. The fitness between such replicates was strongly correlated (ρ = 0.83-0.90 across conditions and p-value < 10-16).
Randomization	We studied fitness of mutations in our samples. All these mutations were introduced using random error prone PCR. We had several internal controls to identify sources of noise and differentiate real fitness changes to those just due to noise.
Blinding	Since, all the vairants were created by random mutagenesis, there was no way of knowing the groups (beneficial versus neutral or deleterious mutations) a priori within the sample. So, by virtue of experimental methodology, all samples were treated the same.

Reporting for specific materials, systems and methods

We require information from authors about some types of materials, experimental systems and methods used in many studies. Here, indicate whether each material, system or method listed is relevant to your study. If you are not sure if a list item applies to your research, read the appropriate section before selecting a response.

Materials & experimental systems

n/a	Involved in the study
☒	☐ Antibodies
☒	☐ Eukaryotic cell lines
☒	☐ Palaeontology and archaeology
☒	☐ Animals and other organisms
☒	☐ Clinical data
☒	☐ Dual use research of concern

Methods

n/a	Involved in the study
☒	☐ ChIP-seq
☒	☐ Flow cytometry
☒	☐ MRI-based neuroimaging
